# The accelerating effect of sugar on wound healing delayed by bevacizumab: A case report

**DOI:** 10.1097/MD.0000000000042539

**Published:** 2025-05-23

**Authors:** Xu Ding, Dongdong Xu, Shi Tang, Tieying Zhang

**Affiliations:** a Department of Oncology, Jilin Province People’s Hospital, Changchun, Jilin, China; b Department of Gastrointestinal Surgery, Jilin Province People’s Hospital, Changchun, Jilin, China.

**Keywords:** adverse effects, bevacizumab, delayed wound healing, local disposition, sugar

## Abstract

**Rationale:**

Bevacizumab, a recombinant humanized monoclonal antibody targeting vascular endothelial growth factor is associated with delayed wound healing. Sugar, a traditional remedy for wound healing, has shown significant efficacy in accelerating recovery. Here, we developed a new strategy for managing bevacizumab-associated delayed wound healing with the topical application of sugar.

**Patient concerns:**

A 42-year-old female with non-small cell lung cancer and brain metastases who underwent emergency surgical treatment for acute appendicitis while on bevacizumab developed delayed wound healing postoperatively.

**Diagnoses:**

At 50 days postoperatively, the wound was still approximately 2 cm, indicating bevacizumab-associated delayed wound healing.

**Interventions:**

The patient had enlarged brain metastases and increased edema due to discontinuation of bevacizumab. Bevacizumab was reintroduced without complete wound healing. Concurrently, topical sugar was applied following routine wound disinfection.

**Outcomes:**

The wound completely healed 21 days after initiating sugar therapy, coinciding with ongoing bevacizumab treatment.

**Lessons:**

The patient’s wound healed completely with the application of topical sugar, even in the context of ongoing bevacizumab therapy, highlighting the efficacy of sugar in managing bevacizumab-induced delayed wound healing.

## 1. Introduction

Antiangiogenic therapy, particularly targeting vascular endothelial growth factor (VEGF) with bevacizumab, is a pivotal strategy in cancer treatment. VEGF, one of the most well-characterized angiogenic factors, stimulates tumor angiogenesis by promoting endothelial cell proliferation and migration,^[1]^ thereby playing a critical role in cancer invasion and metastasis.^[2]^ Bevacizumab, an anti-VEGF monoclonal antibody is increasingly utilized to treat advanced malignancy, while effective in inhibiting tumor vascularization and growth, it also significantly disrupts normal wound healing, a process heavily reliant on angiogenesis.^[3]^

Sugar has been historically utilized in wound care for its multifaceted healing properties.^[4–6]^ It reduces edema, clears necrotic tissue, energizes cells, and forms a protective barrier over wounds.^[7]^ Topical sugar creates a high osmotic pressure, absorbing exudates and approximating wound edges.^[8]^ Crystalline sugar is known to enhance lymphocyte and macrophage activity, bolstering immune response and reducing infection risk.^[9]^ As an optimal medium for fibroblast growth, sugar accelerates wound healing.^[10]^ In addition, sugar forms hydrogen bonds with wound exudate, leading to an increase in the water activity of sugar on the wound surface, creating a moist environment that favors angiogenesis.^[11,12]^ These properties make sugar an effective agent for various wound types, including burns and diabetic ulcers.^[13]^ A povidone-iodine and sugar mixture has shown efficacy in wound closure for cancer patients post radio-chemotherapy.^[14]^ Despite its extensive history in wound care, its role in managing delayed wound healing caused by antiangiogenic therapy, specifically bevacizumab, remains underexplored.

Here we present the first documented case demonstrating that adjunctive application of sugar can accelerate healing of a bevacizumab‑associated non‑healing surgical wound without interrupting anti‑VEGF therapy. This report presents a case of a 42-year-old female with non-small cell lung cancer and brain metastases, treated with bevacizumab for cerebral edema. Despite a prolonged nonhealing postoperative wound, we reintroduced bevacizumab and concurrently applied topical sugar therapy, leading to complete wound healing within 21 days of starting sugar therapy, 71 days after surgery. This case highlights a simple, low-cost approach that effectively balances the demands of oncologic control and tissue repair, offering a novel therapeutic strategy for patients requiring continued antiangiogenic therapy.

## 2. Case presentation

A 42-year-old female with non-small cell lung cancer and brain metastases developed dizziness following 3 sessions of gamma knife radiotherapy. Magnetic resonance imaging revealed a parietal lobe nodule with edema, which indicated radiation-induced cerebral edema. She was treated with bevacizumab and temozolomide for 8 months, alongside intermittent dexamethasone to alleviate brain edema. Five days after the last dose of bevacizumab, the patient developed acute appendicitis. Conservative treatment over the following 2 weeks proved ineffective, necessitating an emergency appendectomy 3 weeks after her last bevacizumab dose.

**Figure 1.**
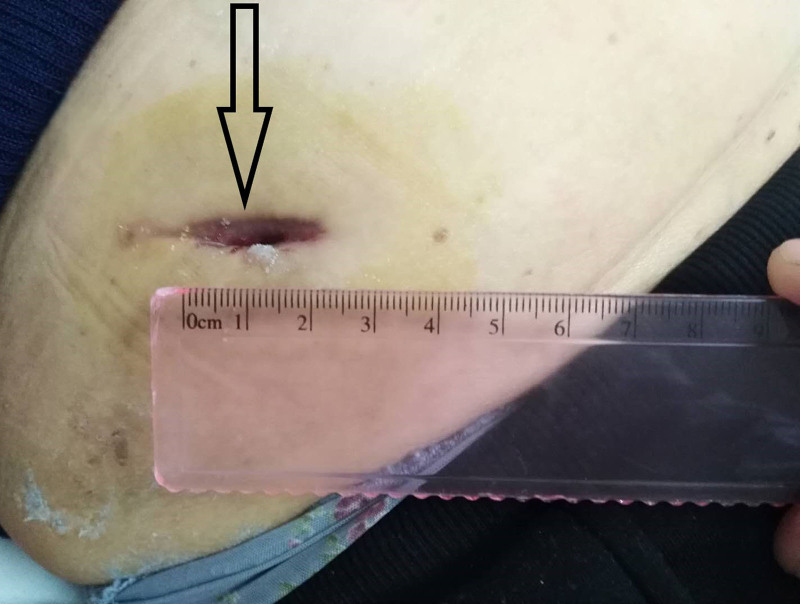
Resumed bevacizumab and treated the 2 cm postoperative wound with sugar therapy at day 50.

**Figure 2.**
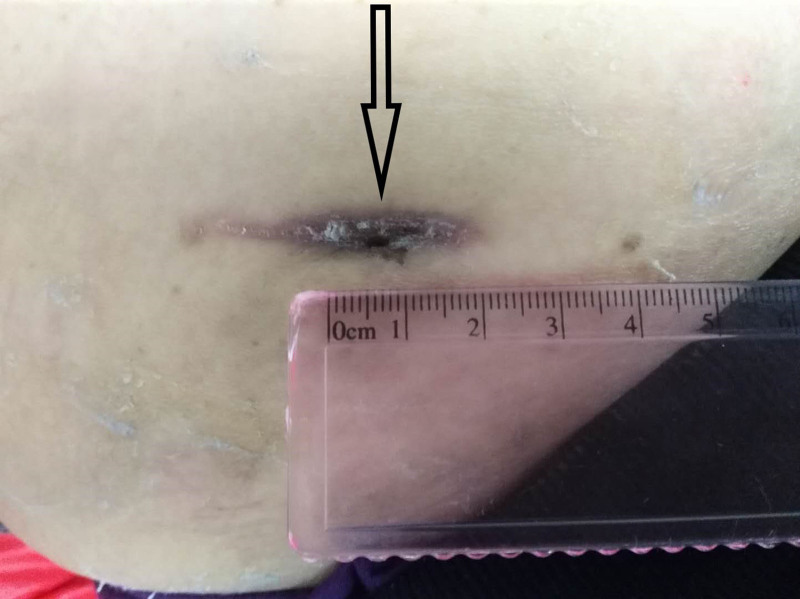
Wound shortened to about 1 cm after 12 days of sugar treatment.

**Figure 3.**
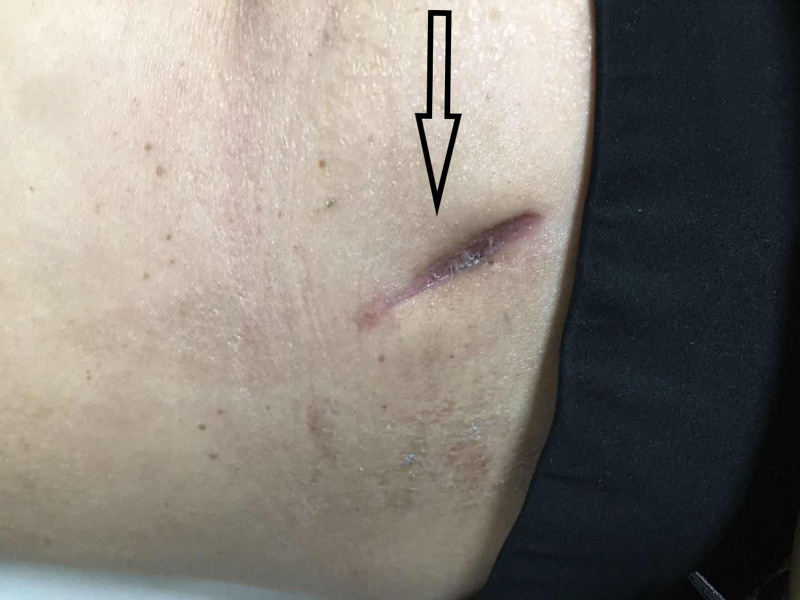
Wound healing was complete after 21 days of sugar treatment.

Postoperatively, despite no bacterial growth, the patient experienced delayed wound healing with pain and discharge (Fig. 1). The patient received standard wound care and continued to use temozolomide instead of bevacizumab. Two cycles of temozolomide therapy resulted in progression of brain metastases and worsening cerebral edema, and bevacizumab was reintroduced in combination with dexamethasone in the absence of wound healing. Given the lack of wound improvement, adjunctive topical sugar therapy was initiated. Granulated white sugar (refined sucrose) was applied directly to cover the wound surface after routine disinfection. The sugar was left in place under a sterile dressing and changed every other day. Remarkably, with continued bevacizumab application, the wound healed completely 21 days after initiation of sugar therapy, 71 days after surgery (Figs. 2 and 3). Subsequent imaging showed stable brain metastases and reduced edema after 2 cycles of combined bevacizumab and temozolomide therapy.

## 3. Discussion

This case underscores the potential of sugar as an adjunct therapy for delayed wound healing induced by bevacizumab. Bevacizumab, by inhibiting VEGF, disrupts angiogenesis – a critical component of normal wound healing – thereby impairing the delivery of inflammatory cells, nutrients, and oxygen to the wound site.^[15]^ The unique properties of sugar, including its ability to reduce infection risk, promote fibroblast proliferation, accelerate granulation tissue formation, create a moist environment that favors angiogenesis,^[7,10–12]^ make it a promising option for enhancing wound healing under challenging conditions.

VEGF plays a crucial role in the proliferative phase of wound healing by mediating endothelial cell activity and promoting coagulation. Consequently, the inhibition of VEGF by bevacizumab can result in significant delays in wound repair by the evidence from both clinical observations and histopathological studies.^[16]^ Previous studies have reported that the incidence of wound complications in brain tumor patients ranged from 10% to 35%.^[17]^ The histopathology of bevacizumab-induced delaying wound healing has been explored in animal models and, to a lesser extent, in human patients. In animal studies, histopathologic examination revealed a reduced number of endothelial cells.^[16]^ Similarly, thinning or even loss of the dermis could result in exposure of subcutaneous fat.^[18]^ Although this is an uncommon complication, its severity often necessitates treatment interruption in clinical settings, underscoring the importance of exercising caution when using bevacizumab.

Current guidelines recommend delaying elective surgery for 4 to 6 weeks post-bevacizumab and resuming treatment only after complete wound healing to minimize wound-healing complications.^[19]^ However, in emergent cases such as acute appendicitis, adherence to these guidelines may not be feasible. In cases of wound-healing complications, bevacizumab should be halted, and wound care measures including regular dressing changes, negative pressure wound therapy, drawing cultures, starting antibiotics, and possibly surgical debridement should be implemented.^[20]^

In this case, the patient underwent an emergency appendectomy for acute appendicitis just 3 weeks after discontinuing bevacizumab. The recent use of bevacizumab significantly contributed to delayed wound healing. During postoperative maintenance therapy, the patient’s brain metastases increased in size and cerebral edema worsened, which was believed to be related to the discontinuation of bevacizumab. Bevacizumab is known to significantly alleviate radiation-induced cerebral edema and improve associated symptoms.^[21]^ The absence of complete wound healing is a contraindication to antiangiogenic therapy, so we decided to reintroduce bevacizumab to inhibit tumor growth and control brain edema. Furthermore, the role of dexamethasone in this situation should not be overlooked. Although corticosteroids are commonly used to reduce edema in patients with brain tumors, subsequent clinical reports have showed that the wound healing complications was potential adverse effect. The combination of bevacizumab and corticosteroids can further compromise the skin’s ability to heal, thereby increasing the risk of delayed wound recovery, and complicating the wound healing process.^[22,23]^ Despite these challenges, the patient’s wound healed completely with the application of topical sugar, even in the context of ongoing bevacizumab therapy.

This case illustrates that topical sugar may serve as an effective adjunct in managing bevacizumab-associated delayed wound healing, even when bevacizumab must be continued due to disease progression. The combination of anti-VEGF therapy and corticosteroids – both known to impair angiogenesis and tissue regeneration – posed significant challenges to wound recovery. Nonetheless, sugar’s multifactorial wound-healing properties, including its osmotic effect, antimicrobial action, and promotion of granulation tissue, facilitated successful healing without discontinuation of anticancer therapy. This outcome underscores the potential of sugar as a low-cost, accessible intervention in oncologic wound care, and highlights the need for individualized management strategies when standard treatment guidelines cannot be followed.

## 4. Conclusion

This case highlights the potential role of sugar in managing bevacizumab-induced delayed wound healing and introduces a novel strategy for addressing this condition. To our knowledge, this is the first documented case highlighting the efficacy of sugar in managing bevacizumab-induced delayed wound healing. Further clinical studies are warranted to validate these findings and explore the broader applicability of sugar therapy in similar contexts.

## Author contributions

**Conceptualization:** Xu Ding.

**Resources:** Dongdong Xu.

**Supervision:** Tieying Zhang.

**Writing – original draft:** Xu Ding.

**Writing – review & editing:** Shi Tang.
